# Comprehensive MR imaging QA of 0.35 T MR‐Linac using a multi‐purpose large FOV phantom: A single‐institution experience

**DOI:** 10.1002/acm2.14066

**Published:** 2023-06-12

**Authors:** Shanti Marasini, Hailei Zhang, Lara Dyke, Mike Cole, Benjamin Quinn, Austen Curcuru, Bruce Gu, Rocco Flores, Taeho Kim

**Affiliations:** ^1^ Department of Radiation Oncology Washington University School of Medicine St. Louis USA; ^2^ MODUSQA London Ontario Canada; ^3^ Department of Biomedical Engineering Washington University School of Medicine St. Louis USA

**Keywords:** large FOV phantom, MR imaging, quality assurance

## Abstract

**Purpose:**

Magnetic resonance‐guided radiotherapy (MRgRT) is desired for the treatment of diseases in the abdominothoracic region, which has a broad imaging area and continuous motion. To ensure accurate treatment delivery, an effective image quality assurance (QA) program, with a phantom that covers the field of view (FOV) similar to a human torso, is required. However, routine image QA for a large FOV is not readily available at many MRgRT centers. In this work, we present the clinical experience of the large FOV MRgRT Insight phantom for periodic daily and monthly comprehensive magnetic resonance imaging (MRI)‐QA and its feasibility compared to the existing institutional routine MRI‐QA procedures in 0.35 T MRgRT.

**Methods:**

Three phantoms; ViewRay cylindrical water phantom, Fluke 76–907 uniformity and linearity phantom, and Modus QA large FOV MRgRT Insight phantom, were imaged on the 0.35 T MR‐Linac. The measurements were made in MRI mode with the true fast imaging with steady‐state free precession (TRUFI) sequence. The ViewRay cylindrical water phantom was imaged in a single‐position setup whereas the Fluke phantom and Insight phantom were imaged in three different orientations: axial, sagittal, and coronal. Additionally, the phased array coil QA was performed using the horizontal base plate of the Insight phantom by placing the desired coil around the base section which was compared to an in‐house built Polyurethane foam phantom for reference.

**Result:**

The Insight phantom captured image artifacts across the entire planar field of view, up to 400 mm, in a single image acquisition, which is beyond the FOV of the conventional phantoms. The geometric distortion test showed a similar distortion of 0.45 ± 0.01  and 0.41 ± 0.01 mm near the isocenter, that is, within 300 mm lengths for Fluke and Insight phantoms, respectively, but showed higher geometric distortion of 0.8 ± 0.4 mm in the peripheral region between 300 and 400 mm of the imaging slice for the Insight phantom. The Insight phantom with multiple image quality features and its accompanying software utilized the modulation transform function (MTF) to evaluate the image spatial resolution. The average MTF values were 0.35 ± 0.01, 0.35 ± 0.01, and 0.34 ± 0.03 for axial, coronal, and sagittal images, respectively. The plane alignment and spatial accuracy of the ViewRay water phantom were measured manually. The phased array coil test for both the Insight phantom and the Polyurethane foam phantoms ensured the proper functionality of each coil element.

**Conclusion:**

The multifunctional large FOV Insight phantom helps in tracking MR imaging quality of the system to a larger extent compared to the routine daily and monthly QA phantoms currently used in our institute. Also, the Insight phantom is found to be more feasible for routine QA with easy setup.

## 1 INTRODUCTION

The radiotherapy treatment planning process requires images with high geometric fidelity and spatial resolution to outline the extent of the disease and plan its accurate treatment.^1^ Magnetic resonance‐guided radiotherapy (MRgRT): integration of MR imaging into the radiotherapy treatment system provides the online visualization of low‐contrast lesions, as well as real‐time target tracking without imparting additional ionizing radiation to the patient. This improves the accurate dose delivery rate and reduces normal tissue complications.^2^, ^3^, ^4^, ^5^, ^6^

MRgRT requires geometric precision of MR imaging in target tracking and/or adaptive planning.^7^ This is necessary to prevent inaccurate dose calculation or improper beam gating. However, one of the challenges with the MRgRT system is dealing with the distortions that are intrinsic to MRI and may alter the accurate representation of anatomical structures. The distortions in MR images are typically divided into two main categories: (a) system‐dependent distortions mainly caused by system imperfections such as inhomogeneities in the main magnetic field (B_0_), gradient nonlinearities, and eddy currents and (b) patient‐induced distortions mainly due to magnetic susceptibility variations and chemical shift effects.^8^ Spatial inaccuracies in MR imaging can lead to target localization uncertainties, which can result in missing or under‐dosing the target or delivering excessive dose to the surrounding tissues.^8^, ^10^ Thus an effective routine quality assurance (QA) program must be in place before clinical treatment. The American College of Radiology (ACR) has launched its standard quality assurance (QA) program for testing MRI system performance which has been later recommended for MRI simulators by AAPM Task Group 284 (TG‐284).^11^ TG‐284 proposes a QA program that includes tests such as spatial fidelity, MRI localization, image quality constancy, and geometric accuracy, all performed at regular intervals. The extent and interval of these measurements vary based on the treatment system, machine setup, and available resources.

Different phantoms are commercially available to accurately quantify the MRI distortions. Each phantom is assigned a distinct label that corresponds to its intention and frequency of use. However, one of the remaining challenges with the MRgRT systems is measuring distortion over a large field of view. The 0.35 T MRgRT system is often used for the treatment of diseases in the abdominothoracic region, with the standard imaging FOV covering a slice area of 350 × 350 mm^2^ and an imaging length of 400 mm.^12^, ^13^, ^14^ The ACR Phantom recommended by TG‐284 is much smaller than the human abdominothoracic region, having an imaging length of 148 mm and a diameter of 190 mm.^11^ Apart from the ACR phantom, other routine QA phantoms have been clinically used for MRI QA tests of MRgRT systems such as the ViewRay cylindrical water phantom and the Fluke 76−907 uniformity and linearity phantom. All of these phantoms cover comparatively small FOVs and require multiple acquisitions for each image metric at different locations to gather a complete data set.^15^, ^16^, ^17^ In addition, some institutes create their own in‐house phantoms covering large FOVs, such as radiation and imaging isocenter alignment phantoms and modular geometric integrity phantoms for MRgRT imaging QA.^18^, ^19^ But, the in‐house phantoms are limited to institutional studies and do not have the full array of test structures provided by the ACR phantom.

Recently, a pre‐release version of the large FOV MRgRT Insight phantom (Modus QA), was studied along with the ACR phantom. The phantom T_1_‐weighted and T_2_‐weighted images were acquired on three different systems; 0.35 T MRgRT, 1.5 T MR‐simulator, and 3.0 T MRI using the ACR scan parameters. The phantoms were scanned using the ACR sequence recommendations to provide a direct comparison with an established QA method. Due to the large field size, the imaging acquisition time of the Insight phantom was nearly double compared to the ACR phantom.^20^ Shorter imaging time helps to improve image QA workflow. In this study, true fast imaging with steady‐state free precession (TRUFI) images were acquired. TRUFI is an ultrafast gradient echo magnetic resonance sequence and is typically used for real‐time imaging during 0.35 T MRgRT treatment due to its short echo and repetition times which reduce the total image acquisition time.^21^

Moreover, the clinical imaging and treatment process in the MRgRT system requires a radiofrequency (RF) coil, which directly impacts the spatial resolution, sensitivity, and uniformity of MRI.^11^ In MRgRT, imaging of abdominothoracic diseases requires flexible torso‐phased array coils. The torso‐phased array coils may undergo significant strain during imaging resulting in coil element failure which can lead to image artifacts with a decreased signal‐to‐noise ratio (SNR). Thus, monthly QA of the individual coil elements of the flexible array coil should be performed to ensure their functionality.^11^ Some institutions perform routine inspections of the phased array coil elements using custom in‐house phantoms, which are not well suited to large‐scale distribution.^22^ This highlights the necessity of a standard coil phantom with a full array of test structures provided by the ACR guidelines. Thus, to achieve all the above‐mentioned purposes, a phantom with a large FOV that can perform the routine QA including system periodic QA and phased array coil QA is imperative.

In this work, we present the single commercial large FOV Insight phantom as the optimal phantom to perform (i) periodic QA on daily and monthly basis and (ii) phased array coil tests on a 0.35 T MRgRT system using the ViewRay TRUFI sequence. Additionally, the QA features of the Insight phantom and its corresponding image analysis software are compared with the ViewRay cylindrical phantom, Fluke 76−907 uniformity and linearity phantom and the in‐house built Polyurethane foam phantom.

## 2 METHODS

### 2.1 Periodic MRI QA tests

**FIGURE 1:**
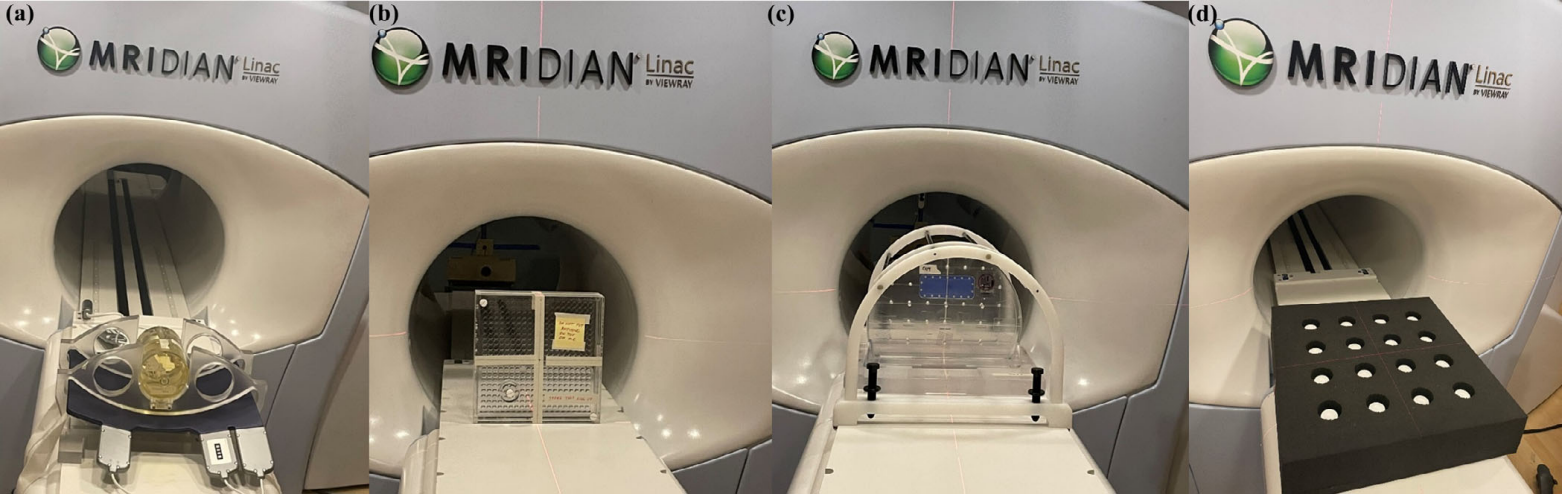
The setup for the (a) ViewRay cylindrical water phantom, (b) Fluke 76−907 uniformity and linearity phantom, (c) MRgRT Insight phantom, (d) Bottle phantom in the 0.35 T ViewRay MRIdian MR‐Linac system.

All measurements were acquired on a 0.35 T ViewRay MRIdian MR‐Linac system (ViewRay, Mountain View, California, USA). Four different phantoms were utilized in this work for the imaging QA (1) the ViewRay cylindrical phantom, (2) the Fluke 76−907 uniformity and linearity QA phantom (hereafter referred to as the Fluke phantom), (3) the Modus QA QUASAR MRgRT Insight QA Phantom (hereafter referred to as the Insight phantom), and (4) the in‐house built Polyurethane foam phantom, which holds bottles of signal producing fluid distributed over a wide field of view (hereafter referred to as the Bottle phantom) (Figure 1). The clinical 3D TRUFI sequence in MRI‐QA mode was used for all tests except for the phased array coil test. The scan parameters used in the study are shown in Tables 1 and 2. All the phantoms were scanned using an integrated body coil with the exception of the phased array coil test.

**TABLE 1:** ViewRay TRUFI sequence MRI scan parameters in MRI‐QA mode for all three phantoms on the 0.35 T MR‐Linac system.

	ViewRay cylindrical phantom	Fluke phantom	Insight phantom
TR (ms)	3.36	3.36	3.36
TE (ms)	1.43	1.43	1.43
Flip angle (°)	60	60	60
FOV (mm)	350 × 350 × 120	350 × 350 × 120	450 × 450 × 120
Matrix	234 × 234 × 40	234 × 234 × 40	300 × 300 × 40
Resolution (mm^3^)	1.5 × 1.5 × 3	1.5 × 1.5 × 3	1.5 × 1.5 × 3
rBW (Hz/Px)	534	534	538
NSA	1	1	1
Acquisition time (s)	19	19	24

FOV, field of view; NSA, number of signal average; rBW, receiving bandwidth; TE, echo time; TR, repetition time.

**TABLE 2:** ViewRay TRUFI sequence MRI scan parameters in MRI‐QA mode for RF coil test phantoms on the 0.35 T MR‐Linac system.

	Bottle phantom	Insight phantom
TR (ms)	147	147
TE (ms)	3.4	3.4
Flip Angle (°)	60	60
FOV (mm^3^)	480 × 480 × 120	480 × 480 × 120
Matrix	256 × 256 × 192	256 × 256 × 192
Resolution (mm^3^)	2 × 2 × 5	2 × 2× 5
rBW (Hz/Px)	300	300
NSA	1	1
Acquisition time (s)	28.2	28.2

FOV, field of view; NSA, number of signal average; rBW, receiving bandwidth; TE, echo time; TR, repetition time.

#### 2.1.1 ViewRay cylindrical phantom

The ViewRay cylindrical phantom (ViewRay Inc., Cleveland, Ohio, USA) shown in Figure 1a was employed for this study as a daily QA phantom. This phantom can be utilized for both imaging and radiation daily QA, but only MR imaging capabilities were explored for this study. The phantom is filled with distilled water for MR imaging. The phantom is inscribed with laser alignment markings which are coincident with the centroid of the active volume in the ionization chamber. The phantom is indexed to the treatment table through two mounting brackets with a cutout for the posterior‐oriented torso phased array coils.

#### 2.1.2 Fluke phantom

The spatial integrity Fluke 76−907 Uniformity and Linearity water phantom doped with 15 Mm CuSO4, (HP Manufacturing, Cleveland, Ohio, USA) is used routinely in our institution as a monthly QA phantom. The routine monthly QA program with Fluke phantom in our facility includes the spatial integrity test in MRI. The phantom has an outer dimension of 330 mm × 330 mm × 102 mm. The phantom consists of two main parts: flood field and grid. The phantom flood field region is filled with degassed water. The phantom contains a 288 mm by 288 mm grid of 397 cylinders spaced approximately 14.5 mm apart (Figure 1b).

#### 2.1.3 Insight phantom

**FIGURE 2:**
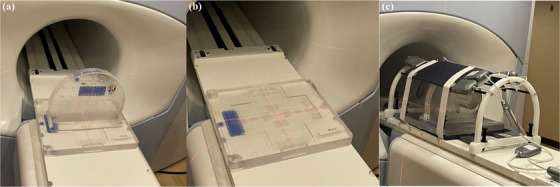
The general structural components for the Insight phantom with (a) the upright plate of the phantom placed on the base plate, (b) the horizontal base plate of the phantom, and (c) the general phantom structure with coil bridge and anterior and posterior torso phased array coils.

The comprehensive QUASAR MRgRT Insight Phantom (Modus QA London, Ontario, Canada), filled with T_1_‐contrast mineral oil was studied for use as routine QA on the MRgRT system using ViewRay scan parameters (Figure 1c). The phantom was designed to serve the QA needs of both the MR imaging and the Linac system, but only the imaging QA was explored in this study. The phantom consists of three main components: an upright plate, a horizontal base plate with its three leveling feet, and a phantom coil bridge (Figure 2). The upright plate is a circular slab 426 mm in diameter and 58 mm in thickness, truncated on the posterior side, and held upright by a 415 mm × 172 mm base. The base plate is a rectangular slab measuring 510 mm × 380 and 46 mm thick. By rotating the upright plate, the phantom was scanned in axial, sagittal, and coronal configurations. The base plate contains a similar arrangement of image quality structures to the upright plate and is intended for coronal imaging. However, the upright plate was used in all three orientations for this experiment in order to ensure that the results were directly comparable. The phantom is inscribed with white lines on the vertical and horizontal structures that are used with the laser landmarking system to ensure the central plane of the phantom coincides with the system isocenter. The Insight phantom has a coil bridge accessory, which was designed to hold the anterior array RF coil over the phantom as shown in Figure 2. For this study, only the integrated body coil was used.

### 2.2 Torso phased‐array receiver coil test

The ViewRay torso phased array coils are made from closed‐cell foam and flexible copper conductors. There are six separate small loops of coil elements, called phased‐array coils. Each coil element has its own amplifier and receiver channel which increases the imaging signal‐to‐noise ratio. With regular bending or wrapping around the patients, these receiver coil elements may undergo significant strain resulting in coil element failures. The phased array coil QA procedure can detect any impaired coil elements. The Bottle phantom and the Insight phantom were compared using the phased array receiver coil test. T_1_‐weighted MRI scan parameters were used to acquire the coronal images for both phantoms. Table 2 shows the scan parameters for the imaging.

#### 2.2.1 Bottle phantom

**FIGURE 3:**
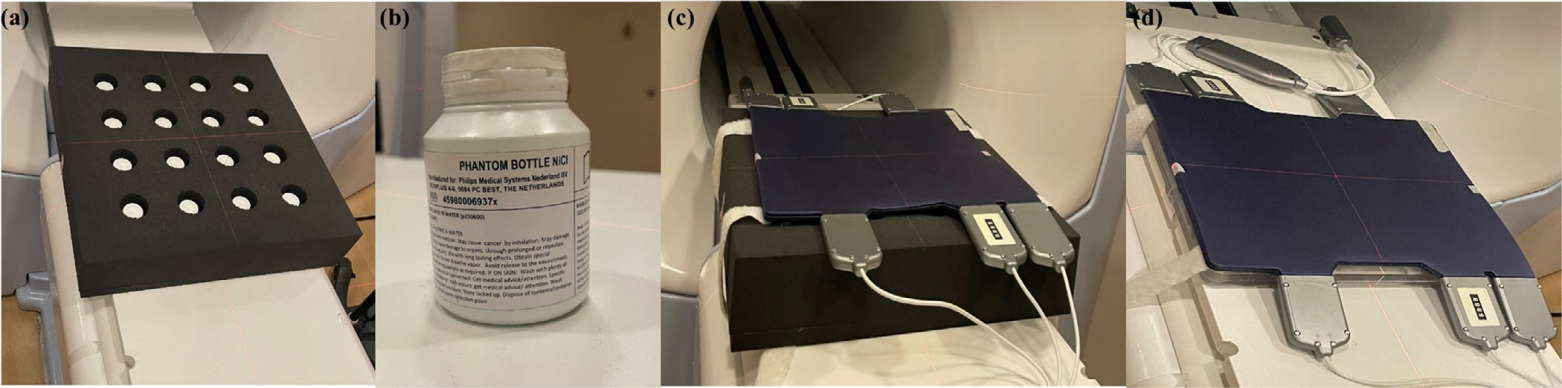
The radiofrequency body coil test with (a) Bottle phantom, (b) NiCl_2_ doped phantom bottle, (c) Bottle phantom with coil under test, and (d) Insight phantom base plate with the coil under test.

The in‐house‐built bottle phantom is a 610 × 610 × 130 mm slab of polyurethane foam and is used in our facility to check for impaired coil elements. The bottle phantom has 16 fiducial holes, each containing a 60 mm diameter bottle filled with NiCl_2_‐doped water, as shown in Figure 3 a‐c. The anterior and posterior torso phased array coils were imaged separately by placing them on top of the phantom.

#### 2.2.2 Insight phantom

The horizontal base plate of the Insight phantom was designed for coronal image acquisition with alignment structures, image quality analysis components, and a geometric distortion grid. As shown in Figure 3 d, the anterior and posterior torso receiver coils were each placed separately above the base plate and scans were performed.

### 2.3 Image analysis

Each phantom measurement was conducted three times to evaluate the stability and reproducibility of the system and methodology using the same imaging parameters. Each measurement's uncertainty was identified by calculating the standard deviation of the corresponding image analysis metrics.

#### 2.3.1 ViewRay cylindrical phantom

**FIGURE 4:**
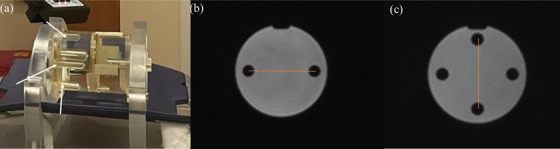
ViewRay cylindrical water phantom with (a) different plugin landmarks in X and Y direction along with ionization chamber. The MR image with two landmark positions in (b) right‐left (RL) positions and (c) anterior‐posterior (AP) positions. The orange lines show the distance measurement between each of the two sets of points. The isocenter variation was observed by comparing the measured value with the reference data of the system.

The daily MR imaging QA program recommended by ViewRay includes the external laser alignment with the imaging plane and basic spatial fidelity test. A virtual isocenter position in the x, y, and z directions was defined using the room laser. The phantom was then aligned to this position using the external scribe marks on the phantom. The position of the phantom in the imaging plane was assessed using four fiducial landmarks at known locations within the phantom (Figure 4). The spatial fidelity was evaluated manually by measuring the distance between the two pairs of fiducials; one pair separated in the RL direction and one in the AP direction (Figure 4b and c).

#### 2.3.2 Fluke phantom

**FIGURE 5:**
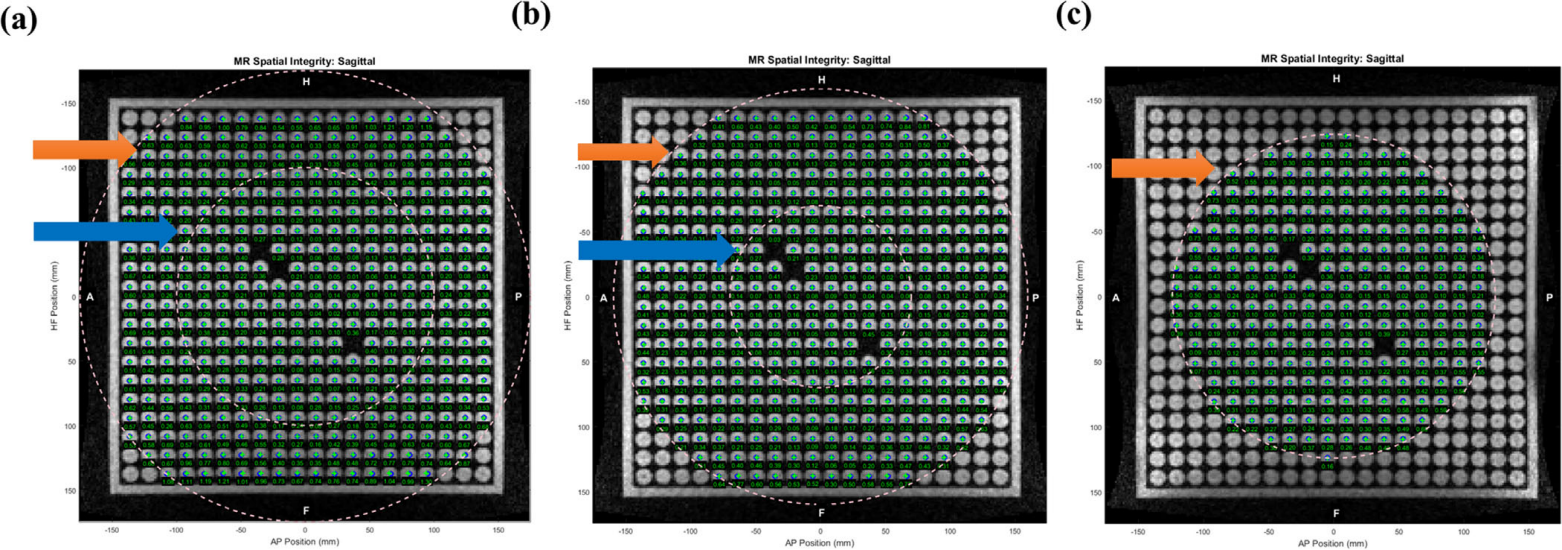
The Fluke phantoms images acquired sagittal position at (a) isocenter, (b) 70 mm, and (c) 125 mm off the isocenter respectively. The circular dots indicate the Uniformity and distortion analysis regions for the phantom. The orange arrow indicates the 175 mm radius region and blue arrow indicates the 100 mm radius region.

The Fluke spatial integrity and uniformity phantom was set up in three orientations; axial, coronal, and sagittal. The axial and coronal images were each acquired at a single location whereas the sagittal images were acquired at five locations: isocenter, 70 and 125 mm to the left, and 70 and 125 mm to the right from the isocenter. This was done to quantify the variation in geometric distortion across the imaging volume, as off‐median sagittal planes that do not intersect the isocenter may be used during treatment (Figure 5). The phantom images were analyzed using ViewRay's SpatialIntegrityAnalysis 2D software. The proprietary software is purpose‐built for the Fluke phantom and performs automatic rotation and translation corrections to account for setup errors. The phantom images were evaluated over two regions: within a radius of 100  and f 175 mm of the phantom center (Figure 5a). The following two tests were performed using the phantom:

(i) Image uniformity: The flood field part of the phantom has a uniform solution, which is used to measure the uniformity in signal intensity of the images. The pixels within a centered geometric area of the phantom, that is, maximum (*P*max) and minimum (*P*min) pixel values were determined by the analysis software. The range (*R*) and mid‐range (*S*) values were calculated using the following equation:
(1) $$R=\left(P\max-P\min\right)/2$$
(2) $$S=\left(P\max+P\min\right)/2$$

The relationship for calculating integral uniformity (U) is U = (1 – (*R*/*S*)) x 100%

Perfect integral uniformity using this relationship is when U = 100%.

For a FOV of 200 mm, the standard imaging uniformity recommended by the AAPM Task Group 1 is 80% or better.^23^

(ii) Spatial integrity: The grid section consists of a 2D array of fiducials of known spacing. The weighted centroid of each circular marker in the grid was compared to the ground‐truth location, labeled by a binary template within two analysis spheres of 100 and 175‐mm radii. The resulting positional error value is reported as a mean and standard deviation. The passing threshold spatial integrity error value of the Fluke phantom is a mean deviation of ≤ 1 mm for a 200 mm diameter spherical volume (DSV) and ≤ 2 mm for a 350 mm DSV.

#### 2.3.3 Insight phantom

**FIGURE 6:**
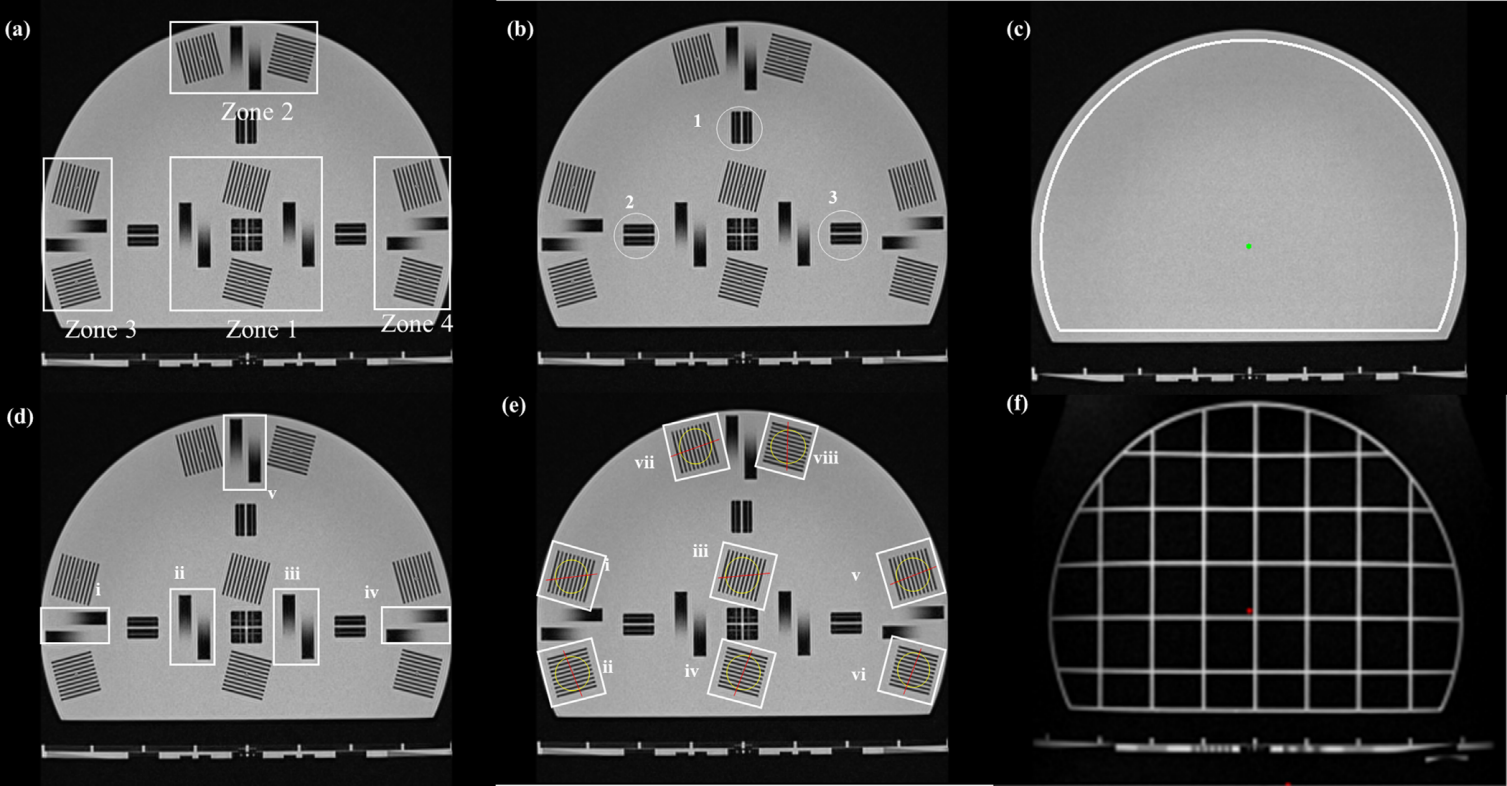
Image analysis regions for the Insight phantom: (a) the four test regions, (b) slice position accuracy test zones, (c) the area that the ROI covers in the flood field for image intensity uniformity, (d) five pairs of ramps for slice thickness test, (e) the high contrast spatial resolution test regions. The red straight line indicates the detected direction of the resolution line pairs, although is not used in the calculation, and the yellow circle denotes the region used to calculate the statistics. (f) The geometric accuracy test region with the geometric accuracy grid.

The signal region of the phantom has a diameter of 400 mm and contains image‐quality structures (Figure 6), a flood field, and a distortion grid. Different sets of tests were performed with the available image quality features present in the phantom. All tests were performed in one scan without phantom movement for each orientation. However, the frequency at which each test needs to be conducted depends on the specific QA program requirement. The image quality features are repeated in four regions of the phantom: the area located near the center of the phantom (zone 1), the anterior region (zone 2), the left region (zone 3), and the right region (zone 4) as shown in Figure 6a. The individual image quality devices are also labeled as zone i to v and i to viii to facilitate reporting of results, as shown in Figure 6. iv and 6. 6 d and e.

All the image quality features of the Insight phantom were compliant with IEC 62464‐1.^24^ The image analysis was performed using the automated software provided with the phantom. All the measurements were repeated three times to calculate the measurement uncertainty. The following five tests were performed:

(i) Phantom positioning: The orientation (translation and rotation) of the phantom relative to the slice axis was detected by the software and reported as an in‐plane offset from isocenter in mm, and a twist around the slice axis in degrees. This test can be used to verify the accuracy of the system positioning lasers (Figure 6b).
(ii) Image uniformity: The image uniformity was measured by creating a uniform flood field region of interest (ROI) covering the full phantom diameter as shown in Figure 6 c. The image uniformity was calculated using the normalized absolute average deviation (NAAD) of the signal within the ROI.
(iii) Slice thickness: The slice thickness accuracy was measured using the five slice thickness ramp pairs, wedge‐shaped signal voids creating image features gradually transitioning from zero signal to full signal (Figure 6d). A line profile was generated along the length of each ramp, covering the transition region, as well as the full‐signal and zero‐signal plateaus at either end of the transition region. Inflection points between the full‐signal plateau sloped transition region and the zero‐signal plateau were used to calculate the length of the transition region. The length of this region divided by the ramp slope provides the slice thickness. The slice thickness of each zone was calculated as the average thickness value from each pair of ramps.
(iv) Spatial resolution: Spatial resolution was evaluated using sets of line pairs rotated by 15° from each of the in‐plane imaging axes. Each imaging zone contains two sets of line pairs aligned at 90 angles to one another allowing for assessment of the frequency and phase encoding direction (Figure 6e).^25^, ^26^ Using the mean and standard deviation of a circular ROI within the line pair region, the software calculated a representative point on the modulation transfer function (MTF) curve.^24^
(v) Geometric distortion: Geometric accuracy was evaluated using the grid section of the phantom by comparing the measured intersection locations with the known phantom dimensions (Figure 6f). The automated software detected the position and orientation of the phantom and generated a truth model of the intersection locations. The software then detected the grid locations in the image and compared them with the truth model to generate a 2D distortion vector field (DVF) representing the in‐plane component of the 3D DVF.^27^ Mean and maximum distortion is reported within and between diameters of 210 , 310 , and 410 mm, relative to the phantom isocenter structure.

## 3 RESULTS

### 3.1 Periodic MRI QA

#### 3.1.1 ViewRay cylindrical phantom

The daily QA ViewRay cylindrical phantom images were analyzed by measuring the distance between the two fiducial landmarks in the horizontal, right‐left (RL), and vertical, anterior‐posterior (AP) positions. The uncertainty of the measurement was estimated by measuring the distances three times. The mean and standard deviation of the measurements were 90.13 ± 0.05  and 90.01 ± 0.02 mm in RL and AP directions, respectively. The reference distance for both the horizontal and vertical fiducials is 90 mm. The deviation in the RL and AP directions was 0.13  and 0.01 mm, respectively, which are within the tolerance specified by TG‐284 (≤ 1 mm within a 200 mm DSV)).^11^

#### 3.1.2 Fluke phantom

The image uniformity values measured using the Fluke phantom were 75.2%, 76.3%, and 75.2% for axial, coronal, and mid‐sagittal planes, respectively. In the sagittal plane, the uniformity value gradually decreased as the phantom moved away from the isocenter (Table 3). The maximum spatial integrity error of 1.17 ± 0.05 was found in the central sagittal image. The mean spatial integrity error for the sagittal plane off‐center acquisitions decreased from 0.45 ± 0.01 at the isocenter to ~0.4 ± 0.02 and 0.25 ± 0.1 at 70 and 125 mm from the isocenter, respectively. This is due to a smaller region of the image plane intersecting the specified DSV, as shown in Figure 5. All the measured spatial integrity error values are within the vendor‐specified tolerance (≤ 1 mm within a 200 mm DSV and ≤ 2 mm within a 350 mm DSV).

**TABLE 3:** Image uniformity and geometric accuracy errors; Mean ± SD and Maximum ± SD values for Fluke phantom.

Scans	Image uniformity (%)	Spatial distortion (mm)	
		Mean	Max
Axial	75.2 ± 0.01	0.31 ± 0	0.85 ± 0.02
Coronal	76.3 ± 0.01	0.3 ± 0.01	0.9 ± 0.05
Sagittal			
(‐125 mm RL)	N/A	0.28 ± 0.1	0.8 ± 0.05
(‐70 mm RL)	60.8 ± 0.2	0.4 ± 0.02	1 ± 0.03
(isocenter)	75.3 ± 0.02	0.45 ± 0.01	1.17 ± 0.05
(+70 mm RL)	56 ± 0.4	0.38 ± 0.03	0.8 ± 0.03
(+125 mm RL)	N/A	0.25 ± 0.1	0.71 ± 0.03

The threshold value for spatial distortion is ≤1 mm for 100 mm and ≤ 2 mm for 175 mm DSV from the isocenter.

#### 3.1.3 Insight phantom

(i) Slice position: The slice position of each plane in all three axes are shown in Table 4. The maximum slice position offset is on the y‐axis with 1.8 ± 0.05 right from the isocenter. The phantom rotation in all three‐axes is within 0.1 degrees.
(ii) Image uniformity: The calculated uniformity (NAAD) values were 92.3 ± 0.04%, 86.1 ± 0.8%, and 75.8 ± 3%, for axial, coronal, and sagittal plane images.
(iii) Slice thickness accuracy: The average slice thickness values were 3.8 ± 0.1 , 4.1 ± 0.1 , and 3.7 ± 0.2 mm for axial, coronal, and sagittal planes as shown in Table 5. The slice thickness measurements were consistently around 25−30% greater than the nominal value of 3 mm. This may be due to the 3D acquisition method used, as Lewis et al.^20^ reported values close to the nominal slice thickness for 2D acquisitions.
(iv) Spatial resolution: The average MTF values were 0.35 ± 0.01, 0.35 ± 0.01, and 0.34 ± 0.03 for axial, coronal, and sagittal planes. The minimum MTF was 0.2 ± 0.01 for the coronal orientation image in zone viii. The IEC 62464‐1 recommended passing threshold value provided in the Insight software for this phantom is 0.8. All MTF values are shown in Table 6 for the frequency and phase encoding directions.
(v) Geometric accuracy: The software analysis produced mean geometric distortion values of 0.66 ± 0.005 , 0.56 ± 0.01 , and 0.84 ± 0.1 mm in axial, coronal, and sagittal plane images, respectively. The vendor‐provided passing threshold value for mean distortion across the full field of view is 2 mm. The maximum geometric error was observed in the peripheral region between the 310 and 410 mm ROI boundaries with mean distortion values of 1.1 ± 0.005 (~0), 0.85 ± 0.005 (~0), and 1.3 ± 0.03 mm for the axial, sagittal, and coronal planes, respectively (Table 7).

**TABLE 4:** The phantom alignment position ± SD and twist ± SD.

Phantom alignment						
	X		Y		Z	
Scan	Position (mm)	Twist (deg)	Position (mm)	Twist (deg)	Position (mm)	Twist (deg)
Axial	0.33 ± 0.05	0	1.93 ± 0.05	0	‐1.5 ± 0	0.1 ± 0.3
Coronal	0.46 ± 0.05	0	0.93 ± 0.05	0.1 ± 0.2	1.5 ± 0.05	0
Sagittal	1.5 ± 0	‐0.1 ± 0.2	1.8 ± 0.05	0	‐1.1 ± 0.05	0

Software‐provided thresholds: Position: ± 5 mm and Twist: ± 1 deg.

**TABLE 5:** Slice thickness ± SD values (mm) at different image locations for the Insight phantom image for three plane orientations.

	Orientation		
Trend (ROI)	Axial	Coronal	Sagittal
zone average	3.8 ± 0.1	4.1 ± 0.1	3.7 ± 0.2
zone i	3.8 ± 0.2	3.8 ± 0.05	4.2 ± 0.7
zone ii	4.1 ± 0.3	4.4 ± 0.1	3.3 ± 0.3
zone iii	4.1 ± 0.1	4.1 ± 0	3.7 ± 0.2
zone iv	4.0 ± 0.2	4.0 ± 0.2	4.1 ± 0.1
zone v	3.2 ± 0.2	4.2 ± 0.2	3.6 ± 0.3

Nominal slice thickness is 3 mm.

**TABLE 6:** Modulation transfer function (MTF) ± SD values for the Insight phantom acquired for phase encoding and frequency encoding direction at different zones.

	Orientation		
Trend (ROIs)	Axial MTF	Coronal MTF	Sagittal MTF
**zone average**	0.35 ± 0.0	0.35 ± 0.01	0.34 ± 0.03
zone i	0.34 ± 0	0.37 ± 0	0.31 ± 0.03
zone ii	0.35 ± 0	0.36 ± 0	0.22 ± 0.02
zone iii	0.42 ± 0	0.4 ± 0	0.46 ± 0.01
zone iv	0.4 ± 0	0.41 ± 0.01	0.44 ± 0. 01
zone v	0.36 ± 0	0.36 ± 0	0.36 ± 0.03
zone vi	0.38 ± 0	0.34 ± 0	0.24 ± 0.01
zone vii	0.35 ± 0	0.33 ± 0.01	0.37 ± 0.01
zone viii	0.35 ± 0.01	0.2 ± 0.01	0.39 ± 0

Uncertainty (SD) values < 0.01 are considered to be 0.

**TABLE 7:** Mean ± SD and Maximum ± SD geometric accuracy error values at different measurement lengths for the Insight Phantom at different imaging orientations.

	Orientation					
	Axial		Coronal		Sagittal	
Area of measurement	Mean	Max	Mean	Max	Mean	Max
Average	0.66 ± 0	1.9 ± 0.03	0.56 ± 0.01	2.26 ± 0.1	0.84 ± 0.1	2.7 ± 0.1
Within 210 mm	0.13 ± 0	0.42 ± 0.02	0.16 ± 0	0.6 ± 0.01	0.16 ± 0.02	0.5 ± 0.03
Within 310 mm	0.22 ± 0	0.55 ± 0.04	0.23 ± 0	0.9 ± 0.03	0.28 ± 0	0.68 ± 0.04
Within 410 mm	0.66 ± 0	1.82 ± 0.04	0.56 ± 0.01	2.26 ± 0.15	0.84 ± 0.01	2.66 ± 0.1
Between 210 and 310 mm	0.31 ± 0	0.55 ± 0.04	0.31 ± 0.01	0.9 ± 0.03	0.42 ± 0.01	0.68 ± 0.04
Between 310 and 410 mm	1.1 ± 0	1.82 ± 0.04	0.85 ± 0.0	2.4 ± 0.15	1.3 ± 0.03	2.5 ± 0.2

The threshold value for mean distortion is 2 mm. Uncertainty (SD) values < 0.01 are considered to be 0.

### 3.2 Phased‐array receiver coil test

**FIGURE 7:**
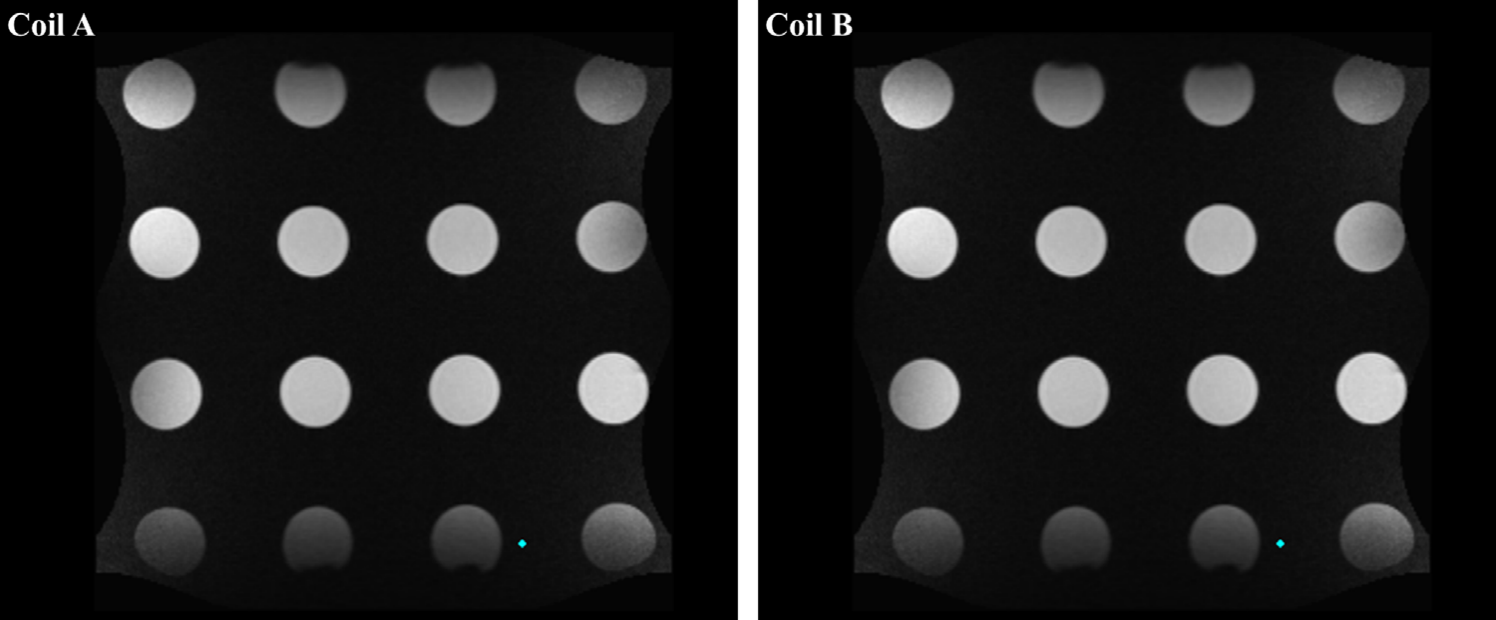
Bottle phantom phased array coil test. The coronal image for the coil elements of a torso array from body coil A and body coil B.

**FIGURE 8:**
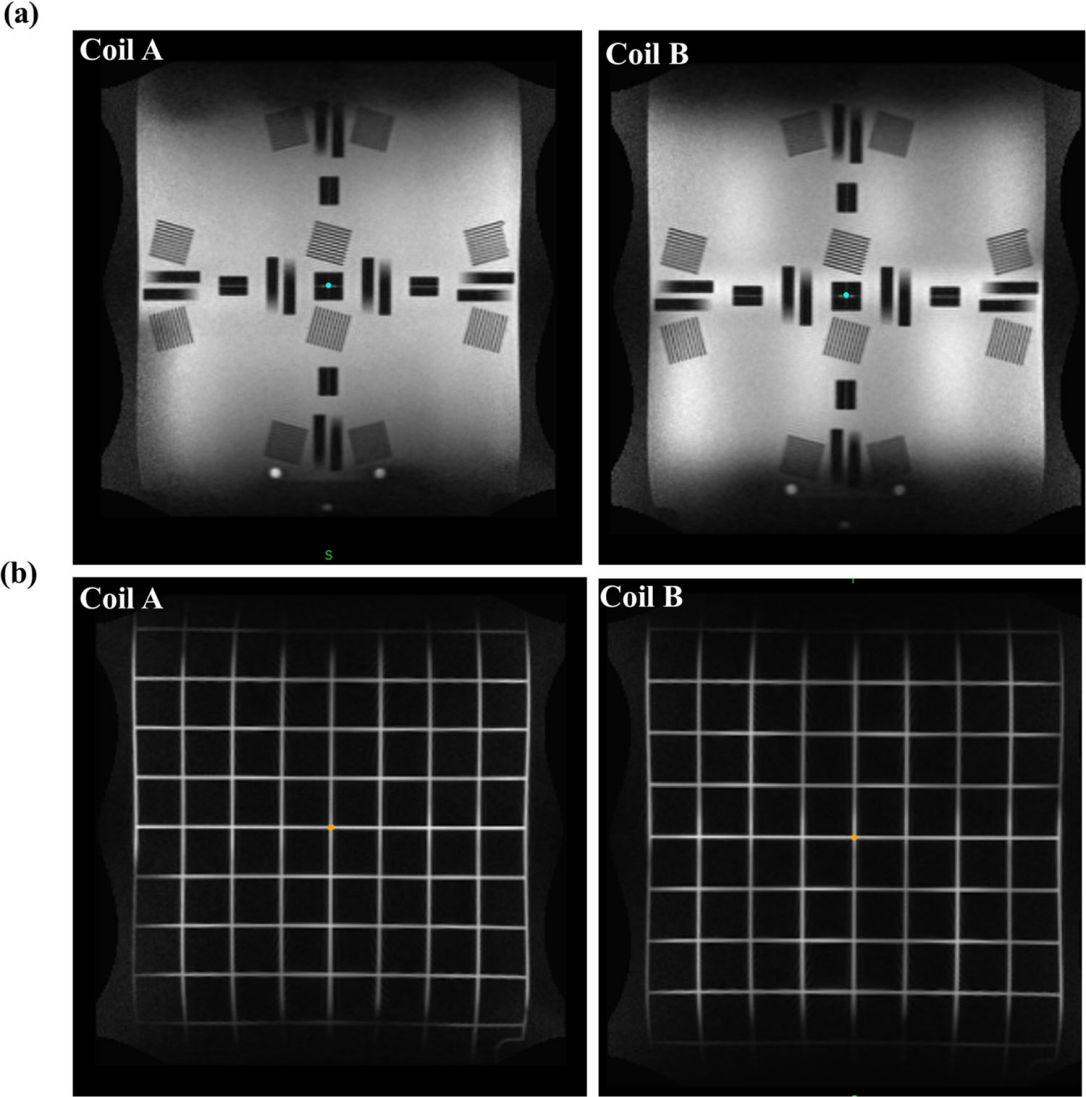
Insight phantom phased array coil test. The coronal image with (a) uniform image quality metrics, (b) grid for the coil elements of a torso array from body coil A and body coil B. The MR images show that all the coil elements are functioning well.

The coronal images of the Bottle phantom and the horizontal base plate of the Insight phantom are shown in Figures 7 and 8. All the coil elements for the anterior and posterior receiver coil have high SNR and uniformity which confirms the functionality of the individual elements. Additionally, the distortion grid pattern of the Insight phantom base provides geometric distortion values across the FOV of the coil. The mean geometric distortion for coil A and coil B was 0.86 ± 0.01 and 0.81 ± 0.03, respectively. The coil elements for the anterior and posterior receiver coils are within the tolerance established for the 0.35 T MRgRT system.^21^

## 4 DISCUSSION

This work presents the clinical experience with the large FOV Insight phantom as a routine QA phantom for a 0.35 T MR‐Linac system. The phantom has an easy setup procedure for daily QA acquisitions. The phantom's automated analysis software was used to analyze the image quality and artifacts based on various image quality metrics. The analysis software promptly provide assesses to the performance of the imaging system. The large diameter of the phantom makes it suitable for evaluating a wider imaging FOV without the need for repositioning. This is especially critical for MRgRT systems where diseases located in the abdominothoracic region are frequently treated, requiring a large imaging area.

The large FOV Insight phantom for routine QA performance on an MRgRT system was investigated previously by Lewis et al.^19^ In the study by Lewis et al., the Insight phantom was compared with the monthly QA ACR phantom, imaged on a 0.35 T MRgRT system and 1.5 and 3.0 T dedicated MRI systems. All the images acquired for the study were T_1_ and T_2_‐ weighted imaging sequences using ACR scan parameters. In the study, the total imaging acquisition time for the T_1_‐weighted imaging scan on 0.35 T MR‐Linac was 3198 s for ACR phantom and 5598 s for Insight phantom, and for the T_2_‐weighted imaging scan was 4608 s for ACR phantom and 8064 s for the Insight phantom. The imaging acquisition time for the Insight phantom is nearly double the time for the ACR phantom, due to the large FOV required for the phantom. In this work, we examined the Insight phantom on 0.35 T MR‐Linac system using the ViewRay clinical TRUFI sequence recommended for ViewRay imaging daily QA along with two other routine QA phantoms, the ViewRay cylindrical phantom and Fluke phantom. The ViewRay and Fluke phantoms are currently used as daily and monthly QA phantoms, respectively, at our institute. The total image acquisition time for each scan was 19, 19, and 24 s for ViewRay cylindrical phantom, Fluke phantom, and Insight phantom, respectively, which is much shorter than imaging sequences using the ACR scan parameters. The slight difference in acquisition between the phantoms is again due to the larger FOV required for the Insight phantom. Thus, incorporating the fast‐imaging TRUFI sequence reduces the total image acquisition time for the Insight phantom immensely making it suitable for daily QA.

The Insight phantom provided similar QA metrics over a large FOV to those provided by both ViewRay cylindrical phantom and Fluke phantom. The Insight phantom automated image analysis software reports the image plane alignment in the two in‐plane directions, along with the in‐plane rotational offset between the phantom and image plane. This in turn leads to more accurate results compared to the manual measurement of the ViewRay cylindrical phantom. The image uniformity values from both the Fluke phantom and Insight phantom were ≥ 80 % in the axial and coronal planes while the value was lower in the sagittal plane for both phantoms. The sagittal plane uniformity value gradually decreased, and could not be analyzed as the phantom moved away from the isocenter, due to the shrinking intersection between the imaging plane and the software‐specified DSV. The uniformity test is further evaluated using the monthly ACR phantom, which is not the part of this study. The geometric distortion results for the Insight phantom showed mean distortions of 0.31 ± 0, 0.31 ± 0.02, and 0.42 ± 0.01 mm in the axial, coronal, and sagittal plane, respectively, within the imaging area of 310 mm, which is similar to Fluke Phantom mean distortion value, that is, 0.31 ± 0, 0.3 ± 0.01, and 0.45 ± 0.01 mm in the axial, coronal, and sagittal plane, respectively. However, the Insight phantom captured a higher geometric distortion of 0.8 ± 0.01 mm in the peripheral region between the 310 and 410 mm ROI boundaries. Furthermore, the imaging analysis results for both Fluke phantom and Insight phantom showed maximum spatial distortion in the sagittal plane. This may be due to higher gradient nonlinearity in the sagittal plane caused by a substantially different design of the Z (superior‐inferior) gradient compared to the X (right‐left) and Y (anterior‐posterior) gradients and/or worse shim in the Z direction in the sagittal plane.^28^

The Insight phantom also allowed slice thickness and spatial resolution to be measured, which is not possible with either the ViewRay cylindrical phantom or the Fluke phantom. The tests were performed in different zones located at the phantom center and edges. The measured slice thickness in all three planes is consistently greater than the nominal value of 3 mm, likely due to the 3D image acquisition technique.^24^ The spatial resolution measurements showed generally lower values in the peripheral zones than at the center of the FOV. This is likely due to increasing magnetic field inhomogeneity and gradient non‐linearity toward the edges of the FOV.^8^, ^28^, ^29^ The results also demonstrate that the resolution in the frequency and phase encoding directions is not necessarily equal. The MTF defined by the line pair structure can detect the changes in the resolution behavior of the system and report it as a trend over time. In general, spatial resolution involves finding the highest spatial frequency at which the MTF curve exceeds a specified threshold value, that is, 0.8. However, IEC 62464‐1 proposes an alternate method appropriate for MRI, where the MTF is evaluated as a single spatial frequency. The MTF values calculated by the Insight phantom software are below the default threshold provided in the software. This can be attributed to the TRUFI sequence of 0.35 T MRI with shorter repetition time and greater bandwidth, as well as the likely presence of a low‐pass reconstruction filter. However, the test remains useful for monitoring changes in image quality, which is the primary function of daily and monthly image QA.

Spatial distortions in MR images arise from both system‐related factors such as gradient non‐linearity, main‐field inhomogeneity, and eddy currents, and also from the magnetic susceptibility and chemical shift effect present in the phantom.^8^ Spatial distortions due to gradient nonlinearities and system eddy current are sequence‐independent and will suffer the same distortion on every image acquired on a particular imaging system. Phantom‐related effects may change due to variations in shimming and differences in the magnetic resonance properties of each phantom. The distortion due to susceptibility effects and main field inhomogeneity was evaluated by using the reverse gradient technique.^29^, ^30^ Half the average distance between locations of each marker in two scans acquired with opposite readout gradient polarities was taken as an upper bound on phantom‐specific susceptibility‐related distortion. The resulting mean distortion values in the frequency encoding direction (y‐axis) were −0.01  and −0.3 mm for the Fluke and Insight phantoms, respectively. The effects on the MR image of such low distortion values are typically less severe than distortions caused by other effects such as gradient nonlinearity.

The phased array coil scans were examined to check for failed coil elements. High SNR and uniformity of all the coil elements were observed, confirming the proper functionality of each coil element. The coil test image for the Insight phantom showed a similar coil element performance to the Bottle phantom coil test image.

The Insight phantom captured the geometric distortion and changes in image quality across the entire planar FOV in single image acquisition for each of the three plane orientations while the Fluke phantom was positioned at multiple sagittal locations to validate the distortion in 2D imaging at offset positions. This is because the Fluke phantom is designed to test the geometric accuracy of the MRI system across different spatial locations. The multiple sagittal image locations help to identify any spatial variations in the distortion across the imaging volume. However, a decreasing area of intersection between the imaging plane and the software‐specified DSV meant that smaller regions of the offset image planes were examined. The Insight phantom could also be used in this manner; however, the base plate is designed to support the phantom in a central location, so the upright plate would likely need to be scanned without the base plate. A large‐FOV 3D distortion phantom is better suited to this task, as it would cover the desired FOV and eliminate the need for multiple scans with phantom repositioning. The Fluke phantom was also able to capture the distortion and uniformity in this manner but does not have the additional image quality features of the Insight phantom.

In addition, the Insight phantom coil bridge structure facilitates the use of an anterior array coil and makes the setup more convenient, and reduces setup variations. Due to the design of the Fluke phantom, the anterior phased array coils could not be used which restricts its scope to QA‐mode only. The Insight phantom automated image analysis software facilitates quick daily QA procedures with more accurate image quality results compared to manual measurement of the ViewRay cylindrical phantom. The automated software detects and corrects the signal intensity gradient across the phantom to improve the reliability of slice thickness measurements. Apart from the above‐mentioned automated image analysis metrics, the software consists of some additional features such as pass/fail/warn mode and performance metrics calculation, which helps to provide quick triage of the system performance in a short period of time and create trending data to help detect any deviations from desired performance.

The large FOV Insight phantom provides an expanded feature set and improved usability and reliability compared to the ViewRay cylindrical phantom and Fluke phantom. However, the phantom had some limitations. First, the phantom base plate which is intended for the analysis of the coronal plane image is subject to higher artifacts at the superior and inferior ends of the FOV due to its large extent. Although the upright plate in the coronal plane can be scanned, the software failed to process the coronal image from the upright plane, and DICOM header modification was needed to facilitate this. Second, due to the large imaging area and relatively thin target structure, accurate alignment of the phantom and imaging space is required. A slight tilt during setup could result in partial volume averaging of the grid and image quality structure region. Third, the Insight phantom image analysis software depends on the image quality. A poor‐quality image or imaging region with blurring or wrapping artifacts may result in the phantom features not being detected correctly. Furthermore, this study was unable to determine the quantitative accuracy of the Insight phantom image analysis software for image analysis metrics. To establish the software's accuracy, additional investigation utilizing a standard phantom with comparable imaging metrics is necessary. Future work with the Insight phantom will be directed towards the integration of radiation delivery QA with imaging QA, which will be an important aspect of the MRgRT system.

Through this work, we established the feasibility of the Insight phantom as a periodic QA on a 0.35 T MR‐Linac system. The QA features of the Insight phantom were compared with the Daily QA ViewRay cylindrical phantom and monthly QA Fluke phantom currently used in our institute. The Insight phantom offers end‐to‐end verification of MR imaging along with radiotherapy as a targeting phantom replacing the need for an additional targeting phantom. The Insight phantom has associated image quality analysis software which provides information on imaging QA parameters including imaging plane alignment, image intensity uniformity, spatial resolution, slice thickness, and geometric accuracy. In addition, the horizontal plate of the Insight phantom offered the ability to monitor the periodic QA features of the receiver phased array body coil including the SNR and uniformity of coil elements.

## 5 CONCLUSION

This work presents the clinical experience with the purpose‐built phantom for a large FOV comprehensive periodic imaging QA. This phantom tracks the MR imaging quality over time at a much higher frequency and a wider range of positions compared to the ViewRay cylindrical phantom and the Fluke phantom which are currently used as daily and monthly QA phantoms in our institute. The automatic image analysis software used in conjunction with the Insight phantom makes it more suitable as a routine daily QA phantom for MRgRT systems.

## AUTHOR CONTRIBUTIONS

Shanti Marasini, Hailei Zhang, Lara Dyke, Austen Curcuru, and Bruce Gu contributed to the data collection, analysis, and writing of the manuscript. Mike Cole, Benjamin Quinn, and Rocco Flores contributed to designing the data analysis system, and analysis tool, and provided technical assistance. Taeho Kim contributed to the data collection, analysis, and supervision of the project. All authors discussed the result and contributed to the final manuscript.

## CONFLICT OF INTEREST STATEMENT

The authors declare no conflicts of interest.

## Data Availability

The data that support the findings of this study are available from the corresponding author upon reasonable request.
